# As Funny as Ever

**Published:** 1867-07

**Authors:** 


### Editorial.

### AS FUNNY AS EVER.

We have a taste for the comic. Those whose actions are droll, while their looks are serious, always afford us solid enjoyment. But a funnier and more enjoyable class of comic actors are those that *feel* intensely serious while performing acts the most ludicrous. The more comic their deeds, the more sanctimonious and self-satisfying are their emotions. They are the happiest mortals in the world, too, except the few who are happy enough to laugh at their drollery.

At the fourth annual meeting of the Odontographic Society of Pennsylvania, "on motion of Prof. McQuillen, the following resolutions were unanimously adopted:

*Resolved*, That the members of this Society, having carefully considered the Code of Ethics adopted by the American Dental Association at the last annual meeting, respectfully decline to accept the same.

*Resolved*, That while pleased to receive *suggestions* from the American Dental Association, the members of this Society do not recognize in the Constitution of the Association the right of that body to dictate terms to the local societies, either with respect to theory, practice, or ethics, or to deny the right of representation to societies which may decline to adopt what has been agreed upon in such directions by that organization."

The first resolution has nothing peculiar in it. If a child "respectfully decline" to eat its dinner, there is an end of the matter. An appetite could not be readily forced into it. If the members of the Odontographic Society after having "carefully considered" the golden rule, should "respectfully decline to accept the same," the American Dental Association proposes to just go on and fulfill its mission, without a thought of a crusade against the Odontographics.

But the second resolution intimates that the members are possibly pleased to receive "*suggestions*" from the American Association.

O don't, Ographics! that's too condescending! Don't you know that your great factotum simply tried his "'prentice hand"

on the American Association, but had set up as a boss workman before he made you?

"Pleased to receive *suggestions*" is good.

But "the members of this Society do not recognize in the constitution of the Association the right of that body to dictate terms to the local societies." That is better; for when did the Association pretend to dictate about anything but its own business, and its own membership. But as the great use of language is the *concealment* of ideas, let us look carefully at this resolution, or we may miss much of the enjoyment that is to be derived from it. The Odontographics "do not recognise in the constitution of the Association the right of that body to deny the right of representation to societies which may decline to adopt" the Code of Ethics. That is a mixture of quotation and paraphrase; but that it is fair and candid may be seen by comparing it with the resolution above. In short, divested of its ambiguity and circumlocution, it means that the American Dental Association has no right to decide who shall compose its membership. It may be claimed that the constitution of the Association does not require a recognition of the Code of Ethics to entitle a local society to representation. And it certainly does not. But that makes no difference. The resolution denies the right of the Association to so amend its constitution, and all this in view of the fact that the constitution of the Association, as all constitutions do, provides for its own amendment.

The constitution, when adopted, defined the conditions of membership; and it would not have been a *constitution* had it failed to do so. This was in accordance with the inherent right of the constituters; and in providing for amendments, they have reserved the right, and made provision for its varied administration.

Of course the Odontographic Society, and all local societies, are entitled to representation, whether they adopt the code or not, but their delegates in subscribing the constitution of the Association, can not "respectfully decline to accept the same." Then what is gained by rejecting the code in the local society? Many members of the Odontographic Society are now members of this Association. These have recognized the code; and its delegates, if it sends any and they discharge the duties delegated to them, will do likewise. Here, then, will be the anomaly of "a house divided against itself"—of part, perhaps a majority, of the members recognizing the code, as members of the Association, while, as members of the local society, they "unanimously" decline to accept the same."

A proposition to amend the constitution is pending; and this proposed amendment *suggests* that local societies shall substantially recognize the Code of Ethics to entitle them to representation. The pending of this may, to some extent, explain the resolutions under consideration. The *Cosmos* report states that Dr. Watt proposed this amendment; but it was proposed by a different man, Dr. Spellman, perhaps, and was simply read by Dr. Watt at his request.

But a little indulgence in biography will throw more light on the resolutions than anything else.

At the Chicago meeting, the Publication Committee was instructed to revise and correct the constitution and by-laws. Several amendments had been adopted; and it was claimed that there were typographical errors. All that any thoughtful person supposed the committee was to do was to correct the print-errors, and insert the amendments in their proper places. But the committee came to Boston with a report, virtually abolishing the code of by-laws, and putting two or three of its sections into the constitution, thus aiming to alter the constitution without the one year's previous notice. One of these *reported* additions to the constitution is as follows:

"Any act of special immorality or unprofessional conduct, committed by a member of this Association, shall be referred to the Committee of Arrangements, whose duty it shall be to thoroughly examine into the case, and report at the next meeting, if the charges be sustained. Whereupon, by vote, the offending member may be reprimanded or expelled."

The chairman of this committee is the author of the Odontographic resolutions. He proposed then to alter the constitution in violation of its own positive stipulations, yet now he introduces a resolution expressing a disbelief in the ability of the Association to alter it in accordance with these stipulations, unless the local societies, or at least the Odontographics are consulted. And the fun of it is that he is in dead earnest all the time.

The same gentleman was a member of the Committee to prepare the Code of Ethics, and he expressed himself to the chairman of the committee as highly pleased with the report; but when it came up for consideration, he was "on general principles opposed to its adoption; as unnecessary for gentlemen, and its enforcement impracticable upon those who were not." And all this occurred although he had tried to force into the constitution but a few hours before, a section making provision for even the expulsion of members for "unprofessional conduct," while the Association had no definite guide or expression as to what unprofessional conduct is. W.

